# Genetic Parameters for Rumination Time, Daily Average Milk Temperature, and Milking Traits Derived from Automatic Milking Systems in Holstein Cattle

**DOI:** 10.3390/ani16030362

**Published:** 2026-01-23

**Authors:** Ali Altınsoy, Hacer Yavuz Altınsoy, Serdar Duru, İsmail Filya

**Affiliations:** 1 Lely International N.V., Cornelis van der Lelylaan 1, 3147 PB Maassluis, The Netherlands; 2 Department of Animal Science, Faculty of Agriculture, University of Uludag, 16059 Bursa, Turkeysduru@uludag.edu.tr (S.D.); ifilya@uludag.edu.tr (İ.F.)

**Keywords:** milkability traits, genetic parameters, rumination, milk temperature, heritability, automatic milking system

## Abstract

Automatic Milking Systems (AMSs) enable the continuous recording of production, milkability, behavioral, and physiological traits, offering new opportunities for genetic evaluation in dairy cattle. This study aimed to estimate variance components and genetic parameters for milk yield-related traits, milking efficiency traits, rumination time (RT), and daily average milk temperature (MTEMP) using AMS-derived data from 1252 Holstein cows. 65,475 weekly records from a single commercial herd were analyzed using repeatability animal models fitted by restricted maximum likelihood. Heritability estimates were moderate to high for milking time (MT) (0.31), milking speed (MS) (0.38), RT (0.30), and MTEMP (0.28), whereas behavioral traits such as number of milking (NoM) (0.26) and number of refused (NoREF) (0.11) showed lower but meaningful heritabilities. Repeatability was highest for MT and MS (0.77 and 0.79), indicating consistent milking performance across repeated records. MTEMP demonstrated clear seasonal variation, increasing in warmer periods and decreasing during colder months, indicating sensitivity to environmental conditions. Genetic correlations among traits revealed both favorable and unfavorable associations; however, several estimates were associated with relatively large standard errors and should therefore be interpreted with caution. The inclusion of MTEMP as a proxy physiological trait derived from AMS data showed measurable genetic variation, although its biological interpretation requires careful consideration. Overall, the results suggest that AMS-derived phenotypes may contribute useful information for genetic studies of functional traits, but the single-herd structure, limited pedigree depth, and data aggregation procedures restrict the generalizability of the findings. Further multi-herd and genomics-based studies are required to validate these results and assess their applicability in breeding programs.

## Simple Summary

Automatic milking systems (AMSs) continuously record large amounts of information on milk production, cow behavior, and physiological indicators. These data offer valuable opportunities to study the genetic background of traits related to milking efficiency, health, and welfare. In this study, data from a Holstein herd managed under AMS were used to evaluate the genetic basis of production, behavioral, and physiological traits, including rumination time and milk temperature. The analyses revealed that these AMS-derived traits are influenced by genetic factors, showing potential for inclusion in breeding programs. Using routinely collected AMS data can help identify cows that are not only productive but also well adapted to automated systems. This study shows that AMS information can support the development of breeding strategies aimed at improving both productivity and animal welfare in modern dairy herds. These results are based on routinely collected AMS data from a single commercial herd, and further multi-herd and large-scale studies are required to confirm and extend these findings.

## 1. Introduction

In dairy cattle breeding, understanding the genetic parameters is crucial for developing selection programs that enhance productivity while maintaining animal health and welfare [1,2].

In recent years, the use of AMS in dairy production has increased rapidly, driving a major transformation in the dairy industry [3,4]. The main motivations for adopting AMS include reducing labor costs, improving cow welfare, and optimizing production processes [5,6]. One of the greatest advantages of AMS is its ability to generate large volumes of consistent and objective data on multiple parameters such as milk yield, milking behavior, rumination time (RT), and milk [7,8,9,10]. These AMS-derived data not only enhance herd management but also provide new opportunities for genetic improvement programs.

Among the key data provided by AMS are milkability traits. Measurements such as milking time (MT), milking speed (MS), and box time are considered essential indicators of milking performance and have been shown to exhibit high heritability [7,11]. With the implementation of AMS, traits such as MS (kg/min), box time (min), milking interval (h), and number of milkings per day (NoM) can now be recorded objectively [7]. Several studies have reported higher heritability estimates for these objectively measured traits, ranging from 0.20 to 0.50 [12,13].

Data obtained from AMS are used not only to evaluate production performance but also to analyze individual cow behavior [3,14]. Number of milkings (NoM), which reflects how frequently a cow voluntarily visits the AMS for milking, has been proposed as a genetically influenced trait associated with both milk yield and cow behavior. In particular, behavioral traits such as milking refusals (MREF) and milking failures directly affect whether cows voluntarily approach the milking robot and their performance during the process [4,15]. These traits are considered to be under genetic control and can be improved through selection [14,16].

Another trait that has attracted increasing attention in recent years is RT. With the development of modern sensor technologies, this trait can now be objectively recorded on a large scale [17,18,19]. RT is closely related to the cow’s health status, stress level, and metabolic activity, and is therefore regarded as an important behavioral indicator of animal welfare [2,20]. RT has also emerged as a critical functional trait associated with metabolic health and methane emissions, thereby gaining importance from an environmental sustainability perspective. Several studies have reported that RT may exhibit positive or negative genetic correlations with milk yield and certain milking traits, and that it can reach moderate to high heritability levels [21,22,23,24]. These findings suggest that RT can serve as both a health indicator and a potential selection criterion. A recent study on Israeli Holsteins estimated the heritability of RT at 0.44 and reported predominantly moderate-to-high genetic correlations with economically important traits such as milk yield, fertility, and longevity [25].

Another emerging trait of interest is milk temperature (MTEMP), which is associated with environmental conditions, metabolic processes, and cow health status. Understanding the heritability of MTEMP and its genetic correlations with milk yield, milking frequency, and metabolic traits may reveal its potential as an indirect selection criterion for disease resistance and thermoregulation [26,27]. MTEMP serves as a reliable indicator of body temperature because the rich blood flow in the udder maintains milk at nearly the same temperature as the cow’s core body temperature [26]. The correlation between MTEMP and body temperature has been reported to range from 0.78 to 0.99 [28,29,30,31]. Although these findings support the use of AMS-recorded MTEMP for identifying cows susceptible to disease, Pohl et al. [27] reported that MTEMP may only partially reflect body temperature in dairy cows.

Estimating the heritability and genetic correlations of behavioral and physiological parameters represents an important approach to be considered in modern dairy cattle genetic evaluations [11,25,32]. In conclusion, the objective of this study was to estimate the variance components and genetic parameters of AMS-recorded traits, including milk yield, milkability traits (MT, MS), behavioral traits (NoM, NoREF), RT, and MTEMP in a Holstein herd located in Turkey. A distinctive aspect of the study is the simultaneous estimation of genetic parameters for MTEMP, RT, and behavioral traits under AMS.

## 2. Materials and Methods

### 2.1. Animals and Definition of Traits

Data used in this study were obtained from a Holstein herd located in the Aegean region of Turkey. The farm operates under Mediterranean climatic conditions. Cows were housed in compost-bedded barns and managed under a Partial Mixed Ration (PMR) feeding system with additional concentrate supplementation through an Automatic Milking System (AMS). All cows were milked using Lely Astronaut A5 units (Lely Industries N.V., Maassluis, The Netherlands).

The traits analyzed in this study were assigned to the day corresponding to the start time of milking. This was particularly important for milkings that began shortly before midnight.

Daily energy-corrected milk (dECM, kg): The dECM was provided by the AMS and calculated using milk fat and protein percentages. Daily values were obtained by summing all 24 h milking records.

Number of milkings (NoM): Represents the number of successful milkings performed by each cow per day.

Number of refused milkings (NoREF): Indicates the number of occasions when a cow approached the AMS unit but was rejected for milking. These events primarily occur due to excessively frequent visits or short intervals between successive visits.

Rumination time (RT, min): Represents the total daily rumination time (min/24 h) recorded by neck-mounted sensors (Lely Qwes HR-LDn, Lely Industries N.V., Maassluis, The Netherlands).

Average milking time (MT, min): Defined as the duration between the start of milk flow and the end of milking for each visit. The average of all milking durations within a day was used. Data recorded in seconds by the AMS were converted to minutes.

Average milking speed (MS, kg/min): Represents the average milk flow rate across all milkings within a 24 h period. The AMS is equipped with sensors capable of recording MS and MT and automatically storing multiple milking-related variables.

Milk temperature (MTEMP, °C): Represents the daily average milk temperature, which was measured by sensors located in the robotic arm during milking.

### 2.2. Data

A total of 184,290 daily records were collected from the AMS software 1.18 between 2021 and 2024. Days in milk (DIM) were coded into weekly periods starting from day 7. To explicitly account for extended lactations, records were retained up to week 65, corresponding to approximately 450 DIM, and observations beyond this point were excluded from the analyses. Thus, extended lactations were not truncated at the conventional 305 DIM but were included up to 450 DIM to capture late-lactation variation, while ensuring a consistent upper bound across cows. Therefore, for all traits, daily values were averaged by week, starting from DIM 7 and matched to the corresponding DIM weeks. The AMS-derived data were summarized by calculating mean values within each DIM class. Converting daily AMS data to weekly averages can reduce phenotypic variance. However, there are a number of technical and biological reasons why researchers still prefer using weekly averages. Daily milk yield from AMS is largely affected by short-term environmental changes (air temperature, shifts in feeding times, temporary stresses, irregularities on lactation days, temporary malfunctions of the milking robot, etc.). Weekly averaging smooths out these fluctuations and provides a more stable phenotype reflecting the animal’s underlying performance rather than daily randomness. AMSs sometimes produce erroneous data (sensor malfunction, incomplete milking records, incorrect animal identification). When using daily data, these errors can cause significant deviations in the model. Taking weekly averages naturally reduces the impact of such outliers on the phenotype. Using mean observations from repeated measurements has been previously applied as a standard procedure [33,34].

For traits that followed a normal distribution, observations exceeding µ ± 3.5σ were excluded from the dataset except for NoREF to remove extreme values likely associated with recording errors, sensor malfunctions, or biologically implausible measurements. The use of the conservative µ ± 3.5σ threshold minimizes the influence of erroneous data while preserving biologically meaningful variation. The minimum and maximum limits used for each trait are presented in Table 1. The applied data range restrictions were as follows: dECM > 3 kg, 190 ≤ RT ≤ 660 min, 1.5 ≤ MT ≤ 10.8 min, 1 ≤ MS ≤ 7 kg/min, 1 ≤ NoM ≤ 5, 0 ≤ NoREF ≤ 8, and MTEMP ≥ 35 °C. The limit values specified here are µ ± 3.5σ, excluding NoREF. Records with NoREF > 8 (2% of all records) were removed, whereas records with NoREF = 0 were retained (12.9% of data; *n* = 8507).

The proportions of records from the first, second, and third or greater lactations were 66.5%, 27.1%, and 6.4%, respectively. The distribution of data across years (2021, 2022, 2023, and 2024) was 4.6%, 10.0%, 26.9%, and 58.5%, respectively. The dataset was balanced across calendar months, with an average of approximately 5000 records per month. About 90% of the data corresponded to the first 45 weeks of DIM, with an average of 1300 records per week.

The data file contained records from 1252 cows, while the pedigree file included 441 sires and 1176 dams. The relatively high number of sires and dams was due to the foundation herd being established with animals imported from the United States. The pedigree depth was limited, with only 111 animals having the longest ancestral path of two generations.

### 2.3. Estimation of Variance Components and Genetic Parameters

Fixed effects were preliminarily explored using GLM to describe data structure; however, final model specification was based on biological relevance and fitted using REML. WOMBAT 2022 was used to estimate variance-covariance components and genetic parameters [35]. Convergence of REML analyses in WOMBAT was assessed by monitoring changes in log-likelihood values, variance component estimates, and first derivatives across iterations, and convergence was assumed when log-likelihood changes were below 10^−6^ and parameter estimates stabilized. Model adequacy was evaluated based on biological plausibility of estimates, absence of boundary solutions, and consistency between single- and two-trait analyses. Although heterogeneous residual variances and autocorrelation across days in milk may be present in daily AMS data, the use of weekly averaged records substantially reduced short-term variability; therefore, homogeneous residual variances were assumed. While random regression or reaction norm models are well-suited for modeling longitudinal AMS data, the present study focused on estimating overall genetic parameters and genetic correlations across traits. Consequently, a repeatability animal model was adopted as a parsimonious and computationally efficient approach for the study objectives.

Initially, analyses were performed in the single-trait repeatability animal model for variance components and then in the two-trait model for genetic correlations. The following is a matrix representation of the model used in both the univariate and bivariate analyses.

y = Xb + Za + Spe + e

In this equation, y refers to the observation values vector for each trait; b denotes the fixed effects vector (milking year = 2021, 2022, 2023, 2024), parity (1, 2, 3+), milking month (January–December), lactation week (from 1 to 65) and a, pe and e stand for direct additive genetic effects (animal), permanent environment effects, and random residual effect, respectively. X, Z, and S are the design and incidence matrices for these effects. A is the numerator relationship matrix. The assumptions used in the analysis are as follows:

$$V(a)\;=\;A\sigma_{a}^{2},\;V(\mathrm{pe})\;=\;I_{pe}\sigma_{pe}^{2},\;V(e)\;=\;I_{n}\sigma_{e}^{2}$$

where V(a), V(pe), and V(e) are variance-covariance matrices, I_pe_ and I_n_ are identity matrices with orders equal to the number of repeat animals and the number of records, $\sigma_{a}^{2}$, $\sigma_{pe}^{2}$, and $\sigma_{e}^{2}$ direct additive genetic variance, permanent environmental (PE) variance, and residual variance, respectively. Dividing these variance components by phenotypic variance, heritability (h^2^) and repeatability (r) were calculated as follows:

$$h^{2}=\frac{\sigma_{a}^{2}}{\sigma_{a}^{2}+\sigma_{pe}^{2}+\sigma_{e}^{2}}$$

$$r=\frac{\sigma_{a}^{2}+\sigma_{pe}^{2}}{\sigma_{a}^{2}+\sigma_{pe}^{2}+\sigma_{e}^{2}}$$

Subsequently, in a series of bivariate analyses based on the same model, genetic (r_g_) correlations using covariance among all traits were derived from covariance component estimates using the following equation:

$$r_{g}=\frac{{Cov}_{a}(y_{1},y_{2})}{\sqrt{{Var}_{a}{(y}_{1})\times {Var}_{a}(y_{2})}}$$

where $r_{g}$ is the genetic correlation between traits $y_{1}$ and $y_{2}$, ${Cov}_{a}(y_{1},y_{2})$ is genetic covariance between traits $y_{1}$ and $y_{2}$, ${Var}_{a}{(y}_{1})$ and ${Var}_{a}(y_{2})$ are genetic variance for the trait $y_{1}$ and $y_{2}$.

Standard errors (SE) of variance components and derived genetic parameters were obtained from the inverse of the average information matrix produced by WOMBAT. The standard error of heritability was approximated using a first-order Taylor series expansion (delta method).

## 3. Results

### 3.1. Descriptive Statistics

Descriptive statistics for all traits are presented in Table 1. The maximum values per milking were 77 kg for dECM and 42 °C for MTEMP. All fixed effects included in the model had significant effects on all traits (*p* < 0.01). As parity increased, dECM, NoM, NoREF, MS, and MTEMP showed increasing trends, whereas RT and MT decreased slightly. The coefficient of determination (R^2^) for MTEMP was the highest among all traits (0.60), indicating that fixed effects explained a substantial proportion of the phenotypic variation.

NoREF showed a highly skewed distribution with a large coefficient of variation (121%), which is typical for AMS-derived behavioral traits. A square root transformation was applied as a sensitivity analysis. Heritability estimates remained comparable to those obtained on the original scale; therefore, the transformation was used only for robustness checking, and all genetic correlations were estimated using the original scale.

**Figure 1:**
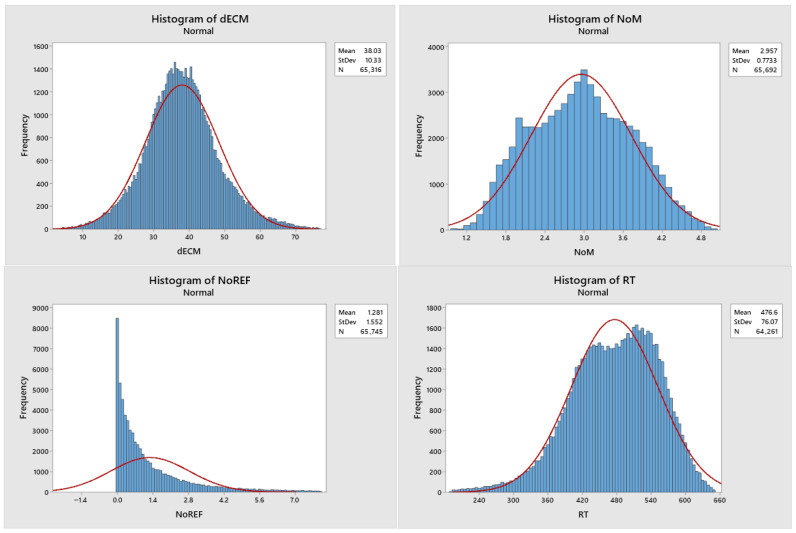

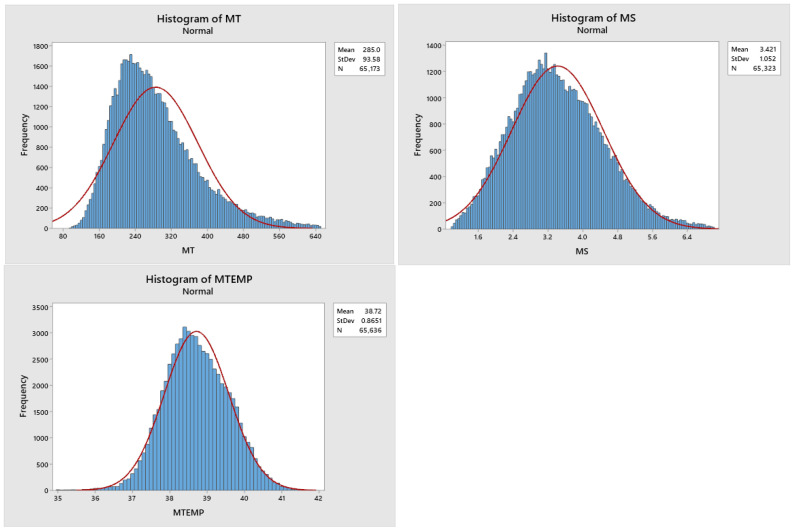
Histograms for all traits. dECM: daily energy corrected milk yield, RT: rumination time, MT: milking time, MS: milking speed, MTEMP: milk temperature, NoM: number of milking, NoREF: number of refusals.

**Figure 2:**
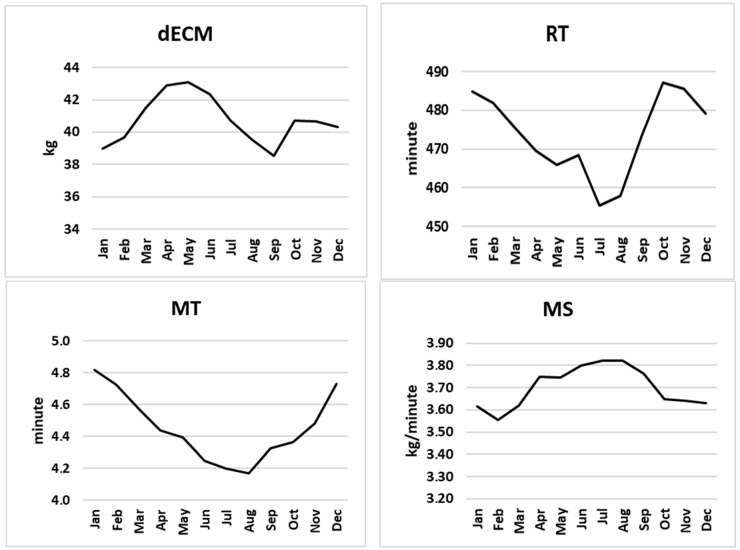

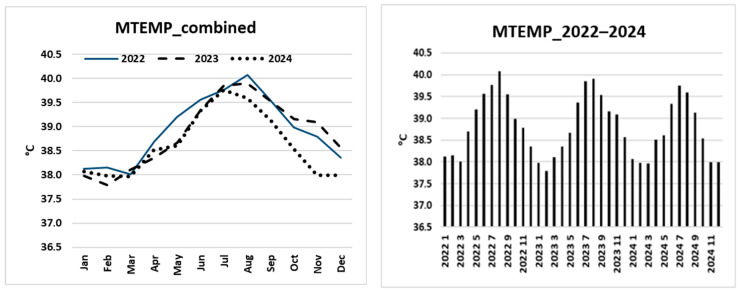
All traits change over the months. dECM: daily energy corrected milk yield, RT: rumination time, MT: milking time, MS: milking speed, MTEMP: milk temperature.

Histograms of the remaining data after removing outlier data are shown in Figure 1, and the annual trends of all traits are illustrated in Figure 2.

### 3.2. Estimation of Heritability and Repeatability

Variance components, heritability, and repeatability estimates are presented in Table 2. Heritability estimates ranged from 0.11 to 0.38. The highest heritability values were observed for MS (0.38), MT (0.31), and RT (0.30), whereas NoREF showed the lowest heritability (0.11). Repeatability estimates ranged from 0.28 to 0.79, indicating moderate to high consistency across repeated records.

The shallow pedigree structure may have reduced the precision of some genetic parameter estimates.

### 3.3. Estimation of Genetic Correlations

Genetic correlations among the analyzed traits are presented in Table 3. Several genetic correlations were estimated with relatively large standard errors (SE > 0.15) and should be interpreted cautiously. Genetic correlation estimates with large standard errors (SE > 0.15) should be interpreted cautiously.

Several genetic correlations showed moderate to high magnitudes; however, most were associated with relatively large standard errors. Therefore, these estimates should be interpreted cautiously and regarded as indicative rather than definitive evidence of genetic relationships.

## 4. Discussion

Seasonal variation was observed for several traits, particularly MTEMP, which increased during summer months in parallel with ambient temperature (Figure 2).

Milk temperature (MTEMP) is closely associated with core body temperature due to the high blood perfusion of the mammary gland and is therefore biologically linked to thermoregulation and metabolic activity. Variation in MTEMP reflects individual differences in physiological responses to heat load and metabolic challenges, which are partly under genetic control. Consequently, estimating the heritability of MTEMP and its genetic relationships with production and milking traits allows evaluation of its potential as an indicator trait for heat stress susceptibility under AMS conditions. However, MTEMP should not be interpreted as a direct measure of core body temperature during individual AMS milking events. Instead, it represents a proxy phenotype capturing short-term physiological variation related to thermoregulatory and metabolic processes, and its interpretation as an indicator of heat stress should therefore be made with appropriate caution.

### 4.1. Heritability and Repeatability

The results indicate notable differences among traits in heritability and repeatability, suggesting that the traits are influenced by genetic and environmental factors to varying degrees.

The heritability estimates obtained in this study ranged from 0.19 to 0.38. The relatively high heritability values observed for some behavioral traits are noteworthy. In particular, the estimate for NoM (0.26) was higher than those previously reported in the literature for similar traits in different populations. Earlier studies have reported varying heritability estimates for NoM. For instance, Carlström et al. [7] found low estimates ranging from 0.02 to 0.07 in Swedish Red and Holstein cows, while Wethal and Heringstad [16] similarly reported a low estimate (0.05) in Norwegian Red cows. Similarly, Nixon et al. [36] estimated the heritability of milking frequency to range from 0.02 to 0.08 in primiparous Holstein cows. These results indicate that genetic variation exists for this trait, but it is generally low in most populations. The slightly higher heritability estimates for NoM observed in the present study may be partly explained by the fact that the analysis was conducted within a single herd, which may have resulted in a smaller estimate of environmental variance. The lack of variation in management, housing, and feeding conditions could have contributed to lower estimates of non-genetic effects, thereby amplifying the relative influence of genetic factors compared with studies involving multiple herds. On the other hand, Aerts et al. [11] reported higher heritability estimates, ranging from 0.15 to 0.32, in Polish Holstein cows, which are more consistent with the findings of the present study and substantially higher than those reported by other researchers. König et al. [34] reported heritability estimates of 0.16, 0.19, and 0.22 in Germany when models were adjusted for milk yield. Based on these findings, they suggested that including production traits in the analysis may influence the genetic variation observed for NoM. Considering the genetic basis of NoM, its relevance in breeding programs is closely linked to the efficiency of robotic milking performance.

Cows with few or no NoREF records are considered preferable, as higher NoREF values reduce the milking capacity of the AMS and are therefore regarded as an undesirable behavioral trait. In the present study, the heritability estimate for NoREF was 0.11, which was slightly higher than some previously reported values. This estimate emphasizes the relatively greater influence of environmental factors on the phenotypic variation in this trait. Wethal and Heringstad [14] reported a heritability of 0.02, while Pedrosa et al. [6] found a value of 0.09 for NoREF. Despite these low heritability estimates, both studies concluded that NoREF is still under genetic influence [6]. Over time, a learning effect is expected; cows generally become more accustomed to using the AMS, particularly regarding refusals [15]. This indicates that cows with more lactations tend to experience fewer refusals in later lactations. However, the opposite pattern was observed in the present study, as NoREF increased up to the third lactation. The low heritability estimates for NoREF and milk yield highlight the importance of environmental and non-additive genetic effects in explaining the variation in these traits. Even for traits with low heritability, selection can still be effective if they are given enough emphasis and are genetically related to other favorable traits. A good example is the genetic improvement achieved in low-heritability traits such as clinical mastitis and ketosis [37].

This study showed that NoM, RT, MT, MS, and MTEMP had moderate heritability estimates, indicating that successful genetic progress can be achieved through direct genetic or genomic selection. Moderate heritability values suggest that these traits can respond directly to selection and may also show reasonable correlated responses in other related traits.

Investigating the genetic background of RT and its relationship with milk yield and milking traits may make it a useful selection criterion in dairy cattle breeding strategies. The results of this study contribute to a better understanding of the genetic variation of RT and its associations with milking traits in dairy cows. In the present study, the heritability and repeatability estimates for RT were 0.30 and 0.59, respectively, with very low standard errors (Table 2). Large variations have been observed in the literature due to differences in breeds, lactation stages, feeding systems, and milking technologies. Sitkowska et al. [2] estimated a low heritability (0.14) and a repeatability of 0.57 for rumination time recorded from AMS in Holstein cows. Similarly, López-Paredes et al. [38] reported heritability and repeatability estimates of 0.17 and 0.45, respectively, for daily rumination time. Lopes et al. [24] found a moderate heritability (0.41) for daily rumination time in Holstein cows, while Moretti et al. [39] reported lower estimates in the same breed, with values of 0.32 in early lactation and 0.34 in mid to late lactation. Moretti et al. [26] also reported heritability estimates of 0.13 for milk yield and 0.32 for rumination time. In another study, Byskov et al. [22] estimated heritability and repeatability for rumination time as 0.33 and 0.75 in a primiparous Holstein research herd, and 0.30 and 0.80 in commercial herds, respectively. Weller and Ezra [25] reported that earlier studies on rumination time were based on smaller datasets and fewer animals, which likely reduced the accuracy of parameter estimates. Using data from more than 70,000 Holstein cows from conventional herds, they estimated a heritability of 0.44 for rumination time. Based on the current selection index that includes rumination time (mean = 536 min, SD = 90 min), the authors predicted an increase of approximately 11 min/day in rumination time after ten years of selection.

MT in cows is determined by milk yield and milking speed, both of which fluctuate across different stages of milking [40]. It has been reported that selecting animals with shorter milking times and higher milk yields can reduce electricity costs and improve the durability of milking equipment [41]. In the present study, the heritability estimate for MT (0.31) was comparable to those reported in some previous studies (0.36–0.43) [14,42,43,44,45]. However, several studies have reported lower heritability estimates ranging from 0.19 to 0.27 [41,46,47,48]. Pedrosa et al. [6] reported that the heritability of milking time ranged from 0.22 to 0.28, remaining low until mid-lactation and then gradually increasing toward the end of lactation. Similarly, heritability estimates for milking time were reported to range from 0.14 to 0.20 in the United States [49] and 0.19 in primiparous Brown Swiss cows in Italy [46]. Compared with these findings, the present study indicates a stronger genetic influence on milking time, suggesting a greater potential for selection and genetic improvement of this trait. Differences in heritability estimates among studies may be attributed to variations in breeds, management systems, data collection methods, and the statistical models used.

In the present study, the highest heritability was found for MS (0.38), which also showed a very high repeatability (0.79). This indicates that MS is a suitable trait for routine genetic evaluation [7,50]. Milking speed is considered a breeding goal of substantial economic value [13,41,51]. Previous studies have shown that selection can potentially improve milking speed [41]. Cows with low milking speed may prolong milking time, reducing workflow efficiency and parlor utilization, whereas cows with higher milking speed can shorten milking duration and contribute to increased profitability [52,53]. On the other hand, slow-milking cows require additional labor, whereas fast-milking cows are more prone to developing udder health problems [13,54]. Therefore, cows with moderate milking speed have been reported to possess healthier teat ends [55]. As milking speed increases, the reduced tension of the teat sphincter may lead to greater susceptibility of the udder to infections [41].

Traits with high repeatability can be measured early in life and still provide reliable predictions of future performance, which is particularly advantageous for making early selection decisions in breeding programs. The relatively high permanent environment variance observed for several AMS-derived traits reflects the strong influence of cow-specific, non-genetic factors that persist across repeated records. Permanent environmental effects encompass stable characteristics such as udder and teat conformation, long-term health and metabolic status, and learned or habitual behaviors related to adaptation to the AMS. Traits such as milking behavior, rumination time, and milk temperature are particularly sensitive to these repeatable influences. In addition, the use of weekly averaged records reduces short-term residual variation, thereby reallocating a larger proportion of the phenotypic variance to the permanent environment component. Similar findings have been reported in previous studies on AMS phenotypes, suggesting that elevated permanent environment variance is an inherent feature of repeatedly recorded behavioral and physiological traits rather than an indication of limited genetic determinism.

Both MT and MS showed high repeatability, indicating that records from the first lactation are sufficient to estimate breeding values for these traits. The repeatability estimate for MT (0.77) found in this study was higher than that reported by Bery et al. [47], while the repeatability for MS (0.79) was slightly lower than the value reported by Carlström et al. [7]. However, these results are comparable to those reported in some studies [12,47,56] and higher than those published in others [13,54,55].

In the present study, the heritability estimate for MS (0.38) was consistent with several previous reports [6,7,11,43,44,57]. However, some studies have reported lower heritability estimates than those found in the present work [13,42,45,48,49,52,53,54,55,58].

Heritability estimates for MS vary depending on measurement methods, breeds, and statistical models. In Canada, a low heritability (0.14) was reported for Holstein cows when a five-point scoring system was used [53], whereas in Irish Holstein herds, a heritability of 0.21 was estimated for average milk flow (kg/min) based on test day records [47]. In studies conducted using AMS in Germany [12,59]. Norway [14], and Sweden [7], heritability estimates for MS ranged from 0.25 to 0.55, depending on whether the data were based on single milkings or 24 h milk yield. For Norwegian Red cows, farmers subjectively scored MS using a three-point scale, and these scores were included in the genetic evaluations for temperament [14]. The heritability of such subjective evaluations varies between 0.16 and 0.25, depending on the breed [60,61,62]. However, when objective measurements obtained from automatic milk meters are used, higher heritability estimates ranging from 0.27 to 0.54 have been observed [42,62].

In this study, the heritability estimate for milk temperature (MTEMP; h^2^ = 0.28) indicates the presence of a moderate genetic component, suggesting that MTEMP may be genetically regulated in addition to being environmentally influenced. Previous studies have reported moderate to strong associations between milk temperature and core body temperature indicators, such as rectal and vaginal temperatures [27,36], supporting its potential relevance as a proxy trait. Consistent with earlier findings [31,33], MTEMP in the present study increased in parallel with ambient temperature, reflecting sensitivity to thermal conditions. Furthermore, the study is limited by the use of data from a single herd, a restricted pedigree structure, and a single management system (Lely A5 AMS), which may constrain the generalizability of the results. These limitations should be considered when interpreting the biological and practical implications of MTEMP as an indicator trait.

### 4.2. Genetic Correlations

The genetic correlations among traits derived from AMS data revealed significant and complex relationships.

The highest genetic correlations involving dECM were found between dECM–NoM (0.26) and dECM–MT (0.38). This pattern is consistent with previous observations; however, the present estimates should be interpreted as indicative rather than evidence of a causal genetic relationship [34,36,63]. In contrast, a low and negative genetic correlation (−0.10) was observed between dECM and RT. Bérat et al. [3] reported a positive genetic correlation (0.34) between rumination time and milk yield, while Lopes et al. [24] observed a higher correlation (0.51). Conversely, Moretti et al. [39] found a slightly negative correlation (−0.04) between rumination time and milk yield during early lactation, and positive correlations (0.13 and 0.12) in mid and late lactation, respectively. Similarly, Atashi et al. [23] reported genetic correlations between rumination time and milk yield ranging from 0.22 to 0.27 for second and first lactations. López-Paredes et al. [38] reported a positive genetic correlation (0.41) between rumination time and milk yield, although the standard error was relatively high (0.75). Taken together, these contrasting results highlight the context-dependency of genetic correlations involving rumination time, particularly with respect to lactation stage and model specification.

Genetic correlations with large standard errors (SE > 0.15) reflect limited estimation precision and should therefore be interpreted cautiously. For this reason, they are discussed only as suggestive trends rather than definitive genetic relationships (Table 3).

In the present study, the genetic correlation between dECM and MS was weak and negative. However, several studies have reported positive and strong relationships [51,60,63]. These differences are likely due to variations in the statistical models used.

The genetic correlation between NoM and NoREF was estimated at 0.84, indicating a strong positive genetic relationship. This suggests that cows with genetically higher milking frequency are more likely to experience milking refusals. Selection aimed at increasing milking frequency could potentially be associated with a higher number of refusals; however, this inference should be made cautiously, given the estimation uncertainty. Aerts et al. [11] reported an average positive genetic correlation (0.70) between milk flow and milk yield when measured on the same day, indicating a strong overall genetic association. However, this correlation varied substantially across different lactation stages and even became negative in later stages.

The moderate negative genetic correlations between NoM–RT (−0.31) and NoREF–RT (−0.33) indicate that cows milked or refused more frequently within a day tended to have shorter rumination times. This association may reflect underlying genetic differences in activity-related traits, although the large SE limits definitive biological interpretation. In other words, more frequent robot visits may be associated with reduced rumination time; however, this relationship should not be interpreted as causal.

The genetic correlation between NoM and MT was estimated at −0.36, indicating a moderate negative genetic relationship. Similarly, Sharipov et al. [64] reported that as milking frequency increased, the duration of each milking session decreased. This relationship may be partly explained by differences in milk accumulation between milkings, although alternative explanations cannot be excluded. However, the authors also noted that excessively long intervals between milkings (>10.5 h) can reduce milk flow efficiency and overall milk yield. These findings highlight the need to maintain an optimal balance between milking frequency and efficiency in dairy cows. Consistently, the present study also demonstrated that cows milked more frequently tended to have shorter milking durations, further supporting the genetic link between milking frequency and efficiency.

The genetic correlation between NoREF and MT was moderate and negative (−0.41), indicating that cows genetically predisposed to longer milking times tend to experience fewer refusals. In contrast, Pedrosa et al. [6] reported a weak positive genetic correlation (0.14) between milking time and refusals.

A strong negative genetic relationship (−0.84) was observed between MT and MS, as flow rate is a direct function of milking time. This reflects a strong inverse genetic association; however, selection responses should be evaluated carefully within a broader breeding objective. In other words, cows with longer milking times tend to have slower milk flow rates. Similarly, Pedrosa et al. [6] reported genetic correlations between milking time and milking speed ranging from −0.65 to −0.85 across lactation, further supporting this relationship.

The genetic correlation between MTEMP and MT was −0.35, indicating a moderate negative genetic relationship. This suggests that cows with longer milking times tend to have slightly lower milk temperatures. The genetic correlation between MTEMP and RT was moderate (0.36), suggesting a shared genetic background between these traits rather than a direct causal relationship. Previous studies have shown that body temperature can increase during milking events [65,66], potentially due to physiological responses such as oxytocin release triggered by tactile stimulation of the udder in AMS. Although Montes et al. [66] reported no phenotypic relationship between rumination and body temperature, rumination is closely linked to digestive activity and metabolic heat production, which may contribute indirectly to temperature variation [67,68]. Therefore, the observed genetic correlation may reflect common regulatory pathways influencing metabolism, activity, and thermoregulation, rather than a direct effect of rumination time on milk temperature. Further physiological and experimental studies are required to disentangle these mechanisms. Overall, these interpretations are presented to provide biological context and should not be regarded as evidence of direct causal mechanisms.

The genetic correlation between MTEMP and MS was moderate (0.59). This suggests a potential genetic association between milking speed and milk temperature, although the underlying biological mechanisms remain unclear. King [69] reported that cows with faster milk flow also had higher body and milk temperatures.

### 4.3. Limitations

Despite all these results, the limitations of this study should also be acknowledged. The findings are based on a single high-yielding Holstein herd and therefore may inflate heritability estimates due to reduced environmental variance. We suggest using genomic relationships in future work because shallow pedigree depth can affect additive genetic variance estimation. Due to a lack of external validation, results may not generalize to other climates, management systems, or breeds. In addition, disease factors that could potentially influence RT were not considered in the analysis. Future studies involving multiple herds and diverse environmental conditions would provide a clearer understanding of the potential use of RT in breeding programs.

## 5. Conclusions

This study provides estimates of genetic parameters for production, milkability, behavioral traits, rumination time, and daily average milk temperature derived from AMS data in Holstein cattle. The results indicate the presence of exploitable genetic variation for several AMS-recorded traits and highlight the substantial contribution of permanent environmental effects. Genetic associations among traits suggest potential relationships between production, milking behavior, and physiological indicators; however, many estimates were characterized by moderate to large standard errors, limiting the strength of biological inferences. The use of single-herd data, shallow pedigree depth, and weekly averaging of daily records may have influenced variance component estimates and restricted the broader applicability of the results. Consequently, the findings should be interpreted as exploratory rather than definitive. Future research incorporating multi-herd datasets, deeper pedigrees or genomic information, and alternative modeling approaches will be essential to confirm these relationships and to clarify the potential role of AMS-derived traits, including milk temperature, in genetic improvement and precision breeding strategies.

## Figures and Tables

**Table 1:** Summary statistics for milk yield traits and milking parameters in Holstein cattle.

Variable	N	Mean	StdDev	CoefVar	Minimum	Median	Maximum
dECM (kg)	65,316	38.0	10.3	27.2	3	37.7	77
NoM (no.)	65,692	2.96	0.77	26.2	1	3	5
NoREF (no.)	65,745	1.28	1.55	121.2	0	0.7	8
RT (min)	64,261	477	76.0	16.0	191	482	650
MT (min)	65,173	4.75	1.56	32.8	1.58	4.47	10.83
MS (kg/min)	65,323	3.42	1.05	30.7	1	3.34	7
MTEMP (°C)	65,636	38.7	0.87	2.2	35	38.7	42

dECM: daily energy corrected milk yield, NoM: number of milkings, NoREF: number of refusals, RT: rumination time, MT: milking time, MS: milking speed, MTEMP: milk temperature, N: number of data, StdDev: standard deviation, CoefVar: coefficient of variation.

**Table 2:** Variance components, heritability, and repeatability for milk yield, milking and behavioral traits, rumination time, and milk temperature.

Trait	$\sigma_{a}^{2}$	$\sigma_{pe}^{2}$	$\sigma_{e}^{2}$	$\sigma_{p}^{2}$	$h^{2}\pm SE$	r±SE	CVa
dECM (kg)	14.11	21.42	37.92	73.45	0.19 ± 0.05	0.48 ± 0.010	0.7
NoM (no.)	0.1359	0.1117	0.2723	0.5199	0.26 ± 0.05	0.48 ± 0.010	3.9
NoREF (no.)	0.2627	0.4052	1.6768	2.3446	0.11 ± 0.03	0.28 ± 0.009	297.9
RT (min)	1641.6	1607.8	2237.1	5486.6	0.30 ± 0.06	0.59 ± 0.010	0.8
MT (min)	0.7490	1.1162	0.5639	2.4291	0.31 ± 0.07	0.77 ± 0.001	7.8
MS (kg/min)	0.4114	0.4417	0.2265	1.0796	0.38 ± 0.08	0.79 ± 0.008	15.0
MTEMP (°C)	0.0993	0.0526	0.1984	0.3504	0.28 ± 0.04	0.43 ± 0.005	104.7

dECM: daily energy corrected milk yield, NoM: number of milkings, NoREF: number of refusals, RT: rumination time, MT: milking time, MS: milking speed, MTEMP: milk temperature, $\sigma_{a}^{2}$: direct additive genetic variance, $\sigma_{pe}^{2}$: permanent environmental variance from the animal, $\sigma_{e}^{2}$: environmental variance, $\sigma_{p}^{2}:$ phenotypic variance, $h^{2}:$ heritability, SE: standard error, r: repeatability, CVa: additive genetic coefficient of variation (σa/mean × 100).

**Table 3:** Genetics correlations with the standard error among investigated traits.

Trait	dECM	NoM	NoREF	RT	MT	MS
NoM (no.)	0.26 ± 0.17					
NoREF (no.)	−0.03 ± 0.19	0.84 ± 0.08				
RT (min)	−0.10 ± 0.19	−0.31 ± 0.17	−0.33 ± 0.17			
MT (min)	0.38 ± 0.18	−0.36 ± 0.16	−0.41 ± 0.15	0.16 ± 0.17		
MS (kg/min)	−0.07 ± 0.18	−0.04 ± 0.17	−0.05 ± 0.17	0.10 ± 0.15	−0.84 ± 0.06	
MTEMP (°C)	0.10 ± 0.16	−0.11 ± 0.15	−0.37 ± 0.15	0.36 ± 0.13	−0.35 ± 0.13	0.59 ± 0.10

dECM: daily energy corrected milk yield, NoM: number of milkings, NoREF: number of refusals, RT: rumination time, MT: milking time, MS: milking speed, MTEMP: milk temperature.

## Data Availability

The raw data supporting the conclusions of this article will be made available by the authors on request.

## Abbreviations

The following abbreviations are used in this manuscript:

AMS	Automatic milking system
dECM	Daily energy-corrected milk (kg)
NoM	Number of milkings per day
NoREF	Number of refused milkings per day
RT	Rumination time (min/day)
MT	Average milking time (min/milking)
MS	Average milking speed (kg/min)
MTEMP	Average milk temperature (°C)
DIM	Days in milk
